# Effect of Heat Stress on Egg Production, Steroid Hormone Synthesis, and Related Gene Expression in Chicken Preovulatory Follicular Granulosa Cells

**DOI:** 10.3390/ani12111467

**Published:** 2022-06-05

**Authors:** Leyan Yan, Mengdie Hu, Lihong Gu, Mingming Lei, Zhe Chen, Huanxi Zhu, Rong Chen

**Affiliations:** 1 Key Laboratory for Crop and Animal Integrated Farming, Ministry of Agriculture and Rural Affairs, Animal Husbandry Institute, Jiangsu Academy of Agricultural Sciences, Nanjing 210014, China; yanleyan198469@126.com (L.Y.); hu_nann011520@163.com (M.H.); 20140036@jaas.ac.cn (M.L.); chenzzju@163.com (Z.C.); 2 Institute of Animal Science and Veterinary Medicine, Hainan Academy of Agricultural Sciences, Haikou 571100, China; nil2008@yeah.net

**Keywords:** heat stress, granulosa cells, progesterone, estradiol, StAR, laying hens

## Abstract

**Simple Summary:**

The debilitating effects of heat stress on poultry production have been well documented. Heat stress already results in severe economic loss worldwide. Regarding the decline in the reproductive performance of heat-stressed hens, the exact mechanisms involved are still unknown. The present study was conducted to elucidate the molecular mechanisms underlying heat-stress-induced abnormal egg production in laying hens. Our results confirmed that laying hens reared under heat stress had impaired laying performance. Follicular granulosa cells cultured in vitro are sensitive to the effects of heat stress, showing an increase in apoptosis and cellular ultrastructural changes. These effects appeared in the form of heat-stress-elevated progesterone, with the increased expression of steroidogenic acute regulatory protein, cytochrome P450 family 11 subfamily A member 1, and 3b-hydroxysteroid dehydrogenase, along with inhibited estradiol synthesis through the decreased expression of follicle-stimulating hormone receptor and the cytochrome P450 family 19 subfamily A member 1. Collectively, laying hens exposed to high temperatures showed damage to granulosa cells that brought about a decline in egg production. This study provides a molecular mechanism for the abnormal laying performance of hens subjected to heat stress, which may help when developing novel strategies to reverse the adverse impact.

**Abstract:**

This study was conducted to elucidate the molecular mechanisms underlying heat stress (HS)-induced abnormal egg-laying in laying hens. Hy-Line brown laying hens were exposed to HS at 32 °C or maintained at 22 °C (control) for 14 days. In addition, granulosa cells (GCs) from preovulatory follicles were subjected to normal (37 °C) or high (41 °C or 43 °C) temperatures in vitro. Proliferation, apoptosis, and steroidogenesis were investigated, and the expression of estrogen and progesterone synthesis-related genes was detected. The results confirmed that laying hens reared under HS had impaired laying performance. HS inhibited proliferation, increased apoptosis, and altered the GC ultrastructure. HS also elevated progesterone secretion by increasing the expression of steroidogenic acute regulatory protein (StAR), cytochrome P450 family 11 subfamily A member 1 (CYP11A1), and 3b-hydroxysteroid dehydrogenase (3β-HSD). In addition, HS inhibited estrogen synthesis in GCs by decreasing the expression of the follicle-stimulating hormone receptor (FSHR) and cytochrome P450 family 19 subfamily A member 1 (CYP19A1). The upregulation of heat shock 70 kDa protein (HSP70) under HS was also observed. Collectively, laying hens exposed to high temperatures experienced damage to follicular GCs and steroidogenesis dysfunction, which reduced their laying performance. This study provides a molecular mechanism for the abnormal laying performance of hens subjected to HS.

## 1. Introduction

High ambient temperatures are harmful to the physiology and production performance of animals, especially laying hens. The optimum temperature range is 19–22 °C for laying hens (the thermoneutral zone); temperatures above this range immediately necessitate the implementation of physiological methods for thermoregulation to cope with heat stress (HS). Hens are particularly vulnerable to HS, owing to their physiological characteristics, including extended egg production cycles up to 75–80 weeks of age [1], feather coverage, and a lack of sweat glands [2,3].

Under heat stress conditions, hens not only show appetite loss, increased water consumption, impaired endocrine systems, and acid-base imbalance [4] but also impairments in their reproductive functions [5,6], such as ovulation and oviposition. It has been reported that HS can lower egg production, egg size, egg quality, and eggshell thickness [7,8,9,10,11]. Reduced feed consumption could be the cause of this impairment in reproductive functions. However, the decreased rate of egg production under high environmental temperatures seems not to be connected with feed intake; Rozenboim et al. [5] suggested that debilitated ovarian functions caused by high environmental temperature might account for reproductive failure. Ovarian follicle development plays an important role in determining poultry laying performance. In the ovarian follicles of laying hens, sex steroids, including estradiol (E2) and progesterone (P_4_), are mainly produced by granulosa cells (GCs) and theca cells [12]. Heat stress can decrease both E2 and P4 levels in the serum of laying hens and impair egg production by reducing the number of follicles [13]. However, the molecular mechanisms underlying the dysfunction of GCs in poultry under heat stress remain unclear.

During follicle development, follicle-stimulating hormone (FSH) and luteinizing hormone (LH) stimulate the cells of the granulosa layer, then the cells begin to express steroid acute regulatory proteins (StAR) and trigger cytochrome P450 cholesterol side-chain cleavage (P450scc, encoded by cytochrome P450 family 11 subfamily A member 1 [CYP11A1]) [14]. The transfer of cholesterol from the outer to the inner mitochondrial membrane is performed by StAR, followed by P450scc transforming cholesterol into pregnenolone, which is then converted to progesterone via 3-hydroxysteroid dehydrogenase (3β-HSD/HSD3B1) [15]. Androgen precursors are synthesized in the thecal cells and are then transported to GCs, where these androgens can be converted to estradiol by aromatase cytochrome P450 (P450arom, encoded by CYP19A1); this process is achieved via the combination of FSH with its receptor (FSHR) [16].

Heat shock proteins (HSPs) have cytoprotective functions against damage that is induced by stress conditions and mediate repair [17]. As the most abundant and sensitive protein of the HSP superfamily, heat shock 70 kDa protein (HSP70) expression is significantly activated in the follicular GCs of hens undergoing heat treatments [18]. However, to date, regarding laying hens experiencing heat stress, the relationship between HSP70 and reduced steroid hormone synthesis in GCs has rarely been discussed and studied.

Taken together, although the debilitating impact of heat stress on laying performance has been well documented, the exact mechanisms involved require further investigation. Therefore, we conducted the present study to determine the effect of heat stress on egg production in laying chickens and its possible mechanisms. To further elucidate the molecular mechanisms of heat-induced ovarian injury, follicular GCs were subjected to high-temperature treatments in vitro, and proliferation and steroidogenesis in GCs were investigated. The expression of estrogen and progesterone synthesis-related genes was also detected.

## 2. Materials and Methods

The experimental procedures were approved by the Research Committee of the Jiangsu Academy of Agricultural Sciences and were carried out strictly in accordance with the Regulations for the Administration of Affairs Concerning Experimental Animals (Decree No. 63 of the Jiangsu Academy of Agricultural Science on 8 July 2014).

### 2.1. Laying Hens, Housing, Heat Stress, and Data Collection

Sixty-four 30-week-old Hy-Line laying hens were housed in two environmentally controlled chambers equipped with a heater, air conditioner, humidifier, dehumidifier, and controller. Each treatment was replicated 8 times and 4 hens were housed per cage for each replicate. Each cage had dispensing nipples and water available ad libitum. The hens were fed ad libitum with a commercial corn and soybean-based diet containing approximately 17.5% CP, 11.2 MJ/kg ME, 3.52% Ca, 0.48% total P, 0.78% Lys, and 0.54% Met.

During the initial 2 weeks, the hens were adapted to the chambers at an ambient temperature of 22 °C and relative humidity of 50%. The chambers were illuminated with white LEDs and the photoperiod included 16 h of light and 8 h of dark. At 32 weeks of age, the hens in the first chamber were continuously maintained under thermoneutral conditions (22 °C) (control group), while for the hens in the second chamber, the temperature was gradually increased (∼2 °C per 1 h) to reach 32 °C and was then kept constant throughout the 14 days (heat stress group). The humidity in both chambers was kept at 50%. Vaccination and medical programs were performed in accordance with common veterinary care practices.

During the day before heat stress (day 0) and the 14-day stress period (day 1–14), eggs were collected and weighed between 9:00 a.m. and 10:00 a.m. each day. Per hen, daily egg production (EP), egg weight (EW), and egg mass (EM) were recorded individually in all cages on a daily basis. EM was calculated using EP and mean EW.

### 2.2. Isolation, Culture, and Treatment of Granulosa Cells

Preovulatory follicular GCs were isolated from hens, according to the method described in a previous study, with minor modifications [19]. In brief, 10 Hy-line brown laying hens (35 weeks of age) were sacrificed by cervical dislocation 2 h after laying eggs, and the ovarian tissue was collected. Follicles larger than 10 mm (preovulatory follicles) were separated and the granulosa layers were separated rapidly. After washing, the granulosa layers were treated with 0.2% collagenase II (Gibco, Gaithersburg, MD, USA) for 15 min at 37 °C. The type-II collagenase was inactivated by adding pre-cooled M199 medium (Thermo Fisher Scientific, Waltham, MA, USA). Through a series of filters, centrifugation, and washing, trypan blue was used for the determination of cell viability. The GCs were cultured in 12-well culture plates, at a density of 1 × 10^6^ or 2 × 10^5^ cells/well at 37 °C, in 5% CO_2_ in air. The basic culture medium used was M199 medium, which was then supplemented with 10% fetal bovine serum (Gibco) and 1% penicillin-streptomycin. After 12 h of incubation, the medium was replaced with fresh medium containing 5% fetal bovine serum, 1% penicillin-streptomycin, 10.0 ng/mL 19-hydroxyandrostenedione (Sigma-Aldrich Corp, St. Louis, MO, USA), and 1 ng/mL FSH (Sigma-Aldrich). The wells were divided into 3 groups thereafter; the control group was continuously cultured at 37 °C, whereas the other two groups were further cultured at 41 °C and 43 °C as the heat treatment group. In other chicken cell cultures and duck granulosa cells, temperatures mimicking heat stress varied between 40 and 45 °C [20,21,22]. Hence, the temperatures of 41 °C and 43 °C in the middle region were chosen in our study. Each group contained three replicates and three separate cultures were conducted. After 12, 24, and 36 h of culture, the cells and culture medium were collected for further analyses.

### 2.3. Flow Cytometric Analysis of Apoptotic Cells

Apoptosis or viability analysis was analyzed with flow cytometry after the combined application of annexin V-FLUOS and propidium iodide, as described in our previous study [23]. Briefly, the GCs were first digested with 0.25% trypsin, then three parallel harvested cells samples were thoroughly washed twice, centrifuged, and resuspended. Subsequently, the cell suspension was supplemented with the binding buffer and annexin V-FITC. The cells were then incubated in the dark at 20–25 °C for 10 min. After centrifugation, the labeled cells were resuspended in a binding buffer containing propidium iodide. Finally, the samples were analyzed using FACS (Becton, Dickinson and Company, Franklin Lake, NJ, USA).

### 2.4. Measurement of Cell Viability

The GCs viability after heat treatment was assessed using a CCK-8 cell viability assay kit (Shanghai QC Bio Science & Technologies Co., Ltd., Shanghai, China) according to the manufacturer’s instructions. After heat treatment for 12, 24, and 36 h, the optical density (OD) of GCs cultured in 96-well plates (2 × 10^3^ cells per well) was measured at 490 nm using a Biotek Eon microtiter plate reader. Cell viability was calculated by the proportion of the absorbance value to that of the control. Blank wells (no cells) and control wells (vehicle alone) were also included. Three separate cultures were performed, and each sample was assayed in triplicate.

### 2.5. Ultrastructure Observation

Ultrastructural observation of GCs cultured in vitro under heat treatment was conducted following the method used in our previous study [23]. Chicken GCs cultured on coverslips were subjected to heat treatment, fixed, dehydrated, dried, coated, and observed under a scanning electron microscope (EVO LS10; Carl Zeiss, Oberkochen, Baden-Württemberg, Germany).

### 2.6. Hormone Secretion Measurements

Estradiol (E2) and progesterone (P4) concentrations in the culture medium were determined by enzyme-linked immunosorbent assay using the ELISA Quantitative Diagnostic Kit for estradiol or progesterone (North Institute of Biological Technology, Beijing, China). The methods used are described in our previous study [24]. The intra- and inter-assay variation coefficients (CV) for E2 were 15%, while the intra- and inter-assay CV for P4 were both under 10%. Each sample was measured in triplicate, and three separate cultures were conducted.

### 2.7. RNA Isolation and Quantitative Polymerase Chain Reaction (q-PCR)

The total RNA from GCs was extracted using a commercial kit (RNAiso Plus, Takara Bio Inc., Shiga, Japan). The qPCR was performed according to the methods in our previous studies [25,26]. Gene-specific primers were designed using Primer 3.0 software and are provided in Table 1. At first, PCR and agarose gel electrophoresis were performed to validate the primers. The results showed that the band of the product was single, and the sizes were the same as expected. In addition, the dissolution curve of q-PCR was a single peak with no primer dimer, and the amplification efficiency is between 90% and 105%. The relative expression levels of the different genes in GCS were calculated using the formula of 2^−ΔΔCT^ [27] and were normalized against the expression levels of β-actin. Each treatment was conducted in triplicate, with three separate cultures being performed.

### 2.8. Statistical Analysis

The mean values of egg production, egg weight, and egg mass were compared between the control and heat treatment groups, using Student’s *t*-test. All other data were analyzed by one-way analysis of variance (ANOVA), with heat treatment as the factor, followed by Tukey’s multiple comparison tests. All data are expressed as the mean ± SEM of three separate experiments. Differences between means were considered to be statistically significant at *p <* 0.05. Statistical analyses were performed using SPSS software (Version 11.0; SPSS Inc., Chicago, IL, USA).

## 3. Results

### 3.1. Effect of Heat Exposure on the Reproductive Activities of Laying Hens

**Figure 1:**
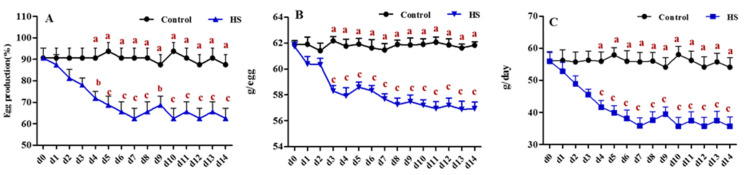
Effect of heat stress on the performance of laying hens. (**A**) Egg production, measured as percentages of laying hens/total hens. (**B**) Average egg weight of laying hens. (**C**) Egg mass, measured as g per egg produced/hen/D, on the day before heat stress (d0) and on each heat-stressed day (d1–d14) in control or stressed (HS) hens. The mean (±SEM) of 8 replicates, with each replicate containing 4 hens. Values with different letters are significantly different (*p* < 0.05).

As shown in Figure 1, the initial egg production (EP), egg weight (EW), and egg mass (EM) were similar between the treatment and control groups before heat exposure (d0). Daily egg production per hen began to drop rapidly in the heat treatment group compared with that in the control group (Figure 1A). As expected, after 4 days of heat exposure, EP dropped from 90.63% in the control group to 71.88% in the heat treatment group, a decrease of 18.75% (*p* < 0.05), and continued to drop during the 2 weeks of heat stress exposure, to a decrease of 62.5% (*p* < 0.01). Heat stress also affected egg weight (Figure 1B). Under heat stress conditions, egg weight declined (*p* < 0.01) in the heat treatment group compared with that in the control group on day 3 and remained low during heat treatment. Consequently, heat stress-exposed hens also had lower egg masses, which dropped to 35.68 g/D (*p* < 0.01) after 2 weeks of heat stress exposure.

### 3.2. Effects of Heat Treatment on Cell Viability and Apoptosis of GCs

**Figure 2:**
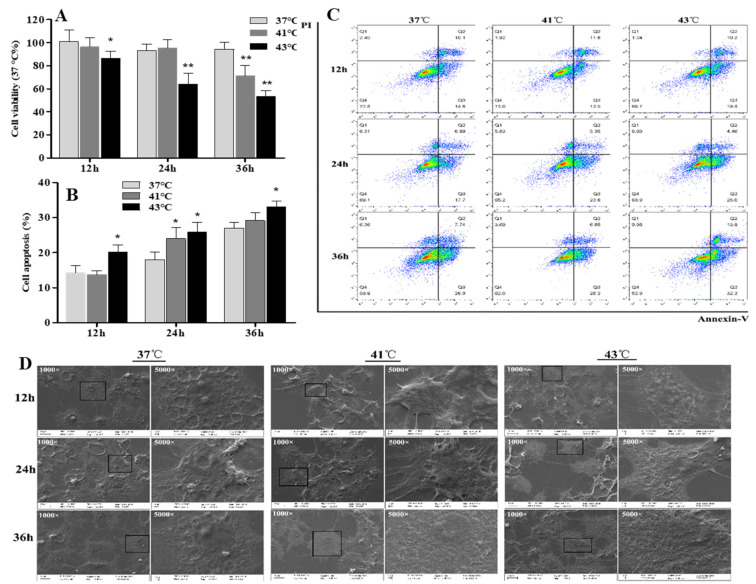
The effect of heat stress on cell vitality and apoptosis of granulosa cells. Primary cultured chicken GC were kept at 41 °C or 43 °C for 12 h, 24 h, or 36 h. (**A**) CCK-8 assay. (**B**,**C**) Flow cytometric analysis, with cells in the area of the lower right corner (Q3) indicating FITC Annexin V-positive cells. (**D**) Scanning electron microscope after heat treatment for 12 h, 24 h, and 36 h. The boxed areas at 1000× were magnified to 5000× and were observed. * *p* < 0.05, ** *p* < 0.01.

Cell morphological observations and FSHR immunohistochemistry staining confirmed that the cultured cells were GCs (Supplementary Figure S1). According to the results in Figure 2A, the viability of follicular GCs after heat treatment at 43 °C for 12 h (*p* < 0.05), 24 h (*p* < 0.01), and 36 h (*p* < 0.01) was significantly lower than that in the control group. The cells exposed to heat treatment at 41 °C showed obviously lower viability than the control group at 36 h (*p* < 0.01). Cell viability was significantly decreased by heat treatment, indicating that high temperatures affected the proliferative ability of GC cells.

To confirm these observations, flow cytometry analysis was conducted. As shown in Figure 2B,C, the cell apoptosis rate in the 43 °C group was significantly increased (*p* < 0.05) at 12, 24, and 36 h when compared to that in the 37 °C group, and the percentage of apoptotic cells in the heat stress-treated group increased with the increase in culture time. However, in the 41 °C treatment group, the number of FITC Annexin V-positive cells was significantly higher (*p* < 0.05) than in the control group (37 °C) after only 24 h of heat treatment (Figure 2B,C).

Cell morphology and ultrastructure were closely related to cell viability. The morphology and ultrastructure of apoptotic cells changed differently. As shown in Figure 2D, when examined using scanning electron microscopy, compared to cells in the control group (37 °C), cells in the heat treatment groups (43 °C group at 24 and 36 h; 41 °C group at 24 h) exhibited condensed cytoplasm around the nucleus, as well as rounded shrinkage. The cell membrane surface also looked rough, and cavitations appeared.

### 3.3. Effects of Heat Treatment on Progesterone and Estradiol Synthesis in GCs

**Figure 3:**
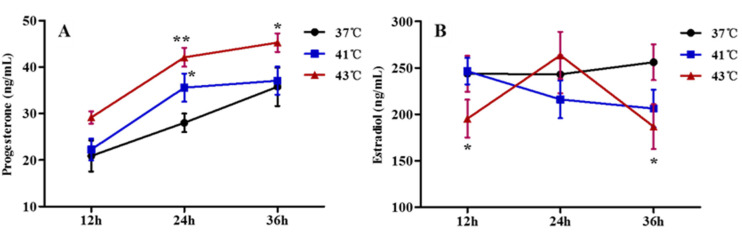
Effects of heat treatment on progesterone and estradiol synthesis in granulosa cells. Primary cultured GCs were kept at 37 °C, 41 °C and 43 °C for 12 h, 24 h, and 36 h, respectively. Progesterone (**A**) and estradiol (**B**) concentrations were analyzed using ELISA methods. * *p* < 0.05, ** *p* < 0.01.

The secretion of P4 and E2 in follicular GCs under high-temperature culture conditions is detailed in Figure 3. In the presence of 1.0 ng/mL FSH and 10.0 ng/mL 19-hydroxyandrostenedione, in comparison with the control group (37 °C), heat treatment at 43 °C for 24 h increased progesterone synthesis by approximately 1.5-fold, compared with that in the control group (*p* < 0.01) (Figure 3A). However, from 24 to 36 h, the increase in progesterone secretion from GCs in the 43 °C group showed a downward trend. In the 41 °C heat-treatment group, the level of P4 was higher (*p* < 0.05; Figure 3A) than that in the control group (37°C group) after only 24 h of treatment.

In contrast, when purified chicken GCs were cultured for 12 and 36 h under a 43 °C heat treatment, E2 concentrations in the culture medium were lower (*p* < 0.05; Figure 3B) than those in the 37 °C control group (Figure 3B). Unexpectedly, E2 levels had a transient elevation after 12 h, while the levels in the 43 °C treatment group were higher than the control group at 24 h, although the difference was insignificant (*p* > 0.05). However, in the 41 °C heat treatment group, E2 concentrations were lower (*p* < 0.05) than those in the 37 °C control group after only 36 h of treatment (Figure 3B).

### 3.4. Effect of Heat Treatment on the Expression of Progesterone- and Estradiol-Synthesizing Enzymes in GCs

**Figure 4:**
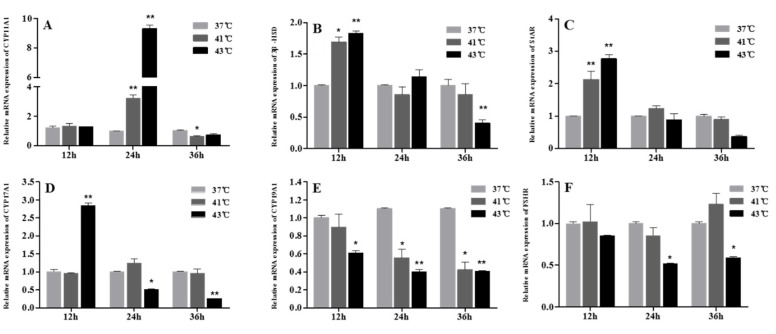
Effect of heat treatment on expression levels of progesterone and estradiol synthesizing enzymes in granulosa cells. (**A**–**F**) CYP11A1, StAR, 3β-HSD, CYP11A1, CYP17A1, CYP19A1, FSHR, CYP19A1, and FSHR mRNA expression levels, respectively. * *p* < 0.05, ** *p* < 0.01.

A high culture temperature significantly affected the mRNA expression of progesterone- and estradiol-synthesizing enzymes. As shown in Figure 4A, CYP11A1 gene expression was strongly upregulated (*p* < 0.01) in the 41 °C and 43 °C groups at 24 h but began to decrease and was lower than that in the cells cultured under control conditions, especially the CYP11A1 gene expression in the 41 °C group at 36 h (*p* < 0.05). The StAR and 3β-HSD mRNA levels were also significantly upregulated after heat treatment at 41 °C (*p* < 0.05) and 43 °C (*p* < 0.01) for 12 h (Figure 4B,C). Conversely, after 36 h of treatment, StAR and 3β-HSD gene expression decreased in the 41 °C and 43 °C heat treatment groups, and the 3β-HSD gene expression in the 43 °C group was significantly lower than that in the control group (Figure 4B,C).

There was no significant difference (*p* > 0.05) in CYP17A1 gene expression between the control and 41 °C heat-treatment groups at each time point (Figure 4D). The gene expression of CYP17A1 in the 43 °C group was significantly higher than in control cells at 12 h (*p* < 0.01). However, at 24 and 36 h, its expression level in the 43 °C group showed a downward trend and was significantly lower (*p* < 0.05) than control. In contrast, the transcription levels of CYP19A1 in the 41 °C and 43 °C groups showed a decreasing trend after heat treatment, and both the transcription levels in the 41 °C (*p* < 0.05) and 43 °C group (*p* < 0.01) were significantly lower than those in normal culture conditions at 24 and 36 h (Figure 4E). Similarly, the 43 °C heat treatment decreased the FSHR mRNA levels in chicken follicular cells after 24 and 36 h (*p* < 0.05, Figure 4F*).*

### 3.5. Effect of Heat Treatment on the Expression Levels of HSP70 and HSP90 in GCs

**Figure 5:**
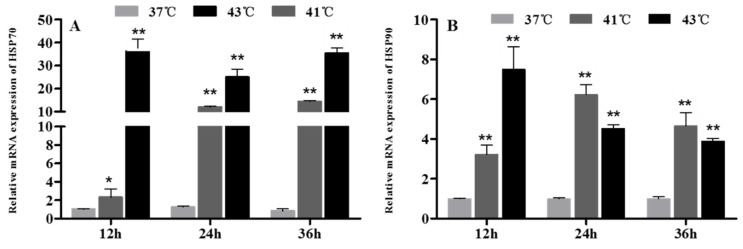
The effect of heat treatment on HSP70 (**A**) and HSP90 (**B**) mRNA expression levels in chicken granulosa cells. * *p* < 0.05, ** *p* < 0.01.

Following heat stress treatments, HSP70 and HSP90 levels were assessed using qPCR. As shown in Figure 5A,B, the expression of both HSP70 and HSP90 showed a sharp increase (*p* < 0.05) after the 41 °C and 43 °C heat treatments for 12, 24, and 36 h. Especially in the cells exposed to 43 °C, the HSP70 mRNA levels were 36-, 25-, and 35-fold those of control after 12, 24, and 36 h of treatment, respectively.

## 4. Discussion

This study clearly shows that high environmental temperatures markedly limit the egg-laying performance of laying hens. These effects were mediated through HSPs and involved steroidogenesis dysfunction in an in vitro chicken granulosa cell culture system. This is probably the mechanism by which heat stress downregulates FSHR and CYP19A1 expression and upregulates StAR expression.

High temperatures above the thermoneutral zone can influence the process of egg formation [28], especially in the case of temperatures exceeding 30 °C. In our study, heat treatment (32 °C) led to negative effects, including a decrease in egg production (25%), egg weight (4.9 g), and egg mass (18.43 g/day), in hens reared under heat stress. Our current results showing decreased laying rates are consistent with previous findings [6,29], which reported that heat stress reduced egg production in heat-stressed laying hens. Other researchers have also shown that exposure to either cyclic or constant heat stress for 1 week could decrease the EW [7,8]. Another study revealed an increase in EW after 2 weeks of heat stress; however, this was probably because the hens were younger in that study, which offset the EW-reducing effect of heat treatment [9]. As a result of the reduction in egg production and weight, egg mass was also negatively impacted by the high ambient temperature. In keeping with our findings, a significant decrease in the egg mass of heat stress-exposed hens was also reported [10]. All these results validate the impaired efficiency of reproductive performance in laying hens suffering from environmental thermal stress.

High temperatures influence hen reproduction, as indicated by oocyte quality, eggshell weight, eggshell quality, fertility, egg production, and egg weight [9,30]; however, despite ovarian follicular development playing a critical role in egg production and the negative effects of heat stress on follicular development, the potential mechanisms remain unknown. In previous studies, compared to hens maintained under thermoneutral conditions (24–26 °C), those exposed to heat stress showed a significant reduction in follicle number [5,13]. Nevertheless, knowledge about the adverse effects of heat stress on the steroidogenic function of ovarian follicles is still limited. This study has addressed this knowledge gap by utilizing a culture system in which preovulatory follicular GCs are present as the main source of estrogen and progesterone secretion.

In the present study, high ambient temperatures decreased the viability and increased the apoptosis of follicular GCs, accompanied by changes in the cellular morphology and ultrastructure. Another study also confirmed that laying hens raised in a high-temperature environment showed increased percentages of TUNEL-positive apoptotic nuclei in mural GCs [13]. Heat-stress-induced apoptosis in follicular cells may indicate a functional deficiency.

The current results showed that heat stress decreased E2 secretion levels from GCs, but increased P4 concentrations in the culture medium. This is consistent with an in vivo study showing that P4 concentration was significantly higher and E2 concentration was lower in the large yellow follicular fluid of stressed hens than in the controls, although both E2 and P4 levels in serum had decreased [13]. Heat stress decreased the E2 concentration in the culture media of duck or bovine GCs but did not alter P4 concentration [22,31]. It is well-known that progesterone is mainly produced by large yellow follicles of laying hens, while small yellow follicles only produced a small amount of P4, and heat stress can cause a decline in the number of large yellow and hierarchical follicles [13]. We speculate that, under heat stress, despite the elevated progesterone production in vitro or in yellow follicular fluid in vivo, the total amount of progesterone production decreased as the large yellow follicle numbers declined. Hence, elevated serum progesterone levels are not typically seen.

To better understand the underlying molecular regulatory mechanisms, several differentially expressed transcripts implicated in steroidogenesis (e.g., StAR and 3β-HSD) were investigated. The basic enzymatic pathways that catalyze cholesterol into progesterone, including P450scc, 3β-HSD, and StAR, are often the main rate-limiting proteins in progesterone formation [32]. In the present study, the mRNA expression levels of StAR, 3β-HSD, and CYP11A1 in GCs increased early in the heat stress groups, which is in agreement with previous findings that heat stress also upregulated StAR and 3β-HSD expression in duck follicular cells [22]. We also found that StAR, 3β-HSD, and CYP11A1 gene expression decreased after 36 h of heat exposure. In bovine GCs cultured under high temperatures, the mRNA expression of CYP11A1 and StAR also decreased [31]. This discrepancy may be related to differences in the effects of heat stress on steroidogenic gene expression in different cell environments. In addition, CYP17A1 gene expression initially increased, but finally decreased. CYP17A1 encodes P450c17 and is in charge of the conversion of progestin to androgen [33]. Heat stress downregulates CYP17A1 expression, which may aggravate the accumulation of progesterone. Based on the above results, the increase in StAR, 3β-HSD, and CYP11A1 expression and the consequent over-secretion of progesterone may be a compensatory scheme in response to the unmet steroid production demanded by ovarian cells [34]. Unexpectedly, 36 h of heat treatment decreased StAR, CYP11A, and 3β-HSD expression in GCs, whereas progesterone production continually increased. In fact, the increase in progesterone secretion from GCs in the high-temperature culture group was less than that in the normal cells after 24 h of culture, which indicated that the rate of progesterone synthesis began to decrease slowly. This was closely related to the downregulation of StAR, CYP11A, and 3β-HSD expression at this time point. The reasons for this progesterone synthesis/secretion pattern after heat stress are unclear; it may be associated with higher apoptosis and the cell function diminishing as this process progresses, but future experiments are necessary to verify this.

As heat stress influences E2 synthesis in GCs, we examined the expression of genes encoding the proteins essential for E2 production. Our results indicated that heat stress decreased FSHR and CYP19A1 gene expression in GCs. As is well known, FSH, FSHR, and CYP19A1 are intimately connected to estradiol production in GCs [35]. Heat stress also inhibits estrogen synthesis in duck GCs through the reduction of CYP19A1 expression [22]. In the present study, reduced estrogen concentrations in the culture medium of heat treatment cells were accompanied by decreasing FSHR and CYP19A1 transcription levels, which locally implies that heat stress potentially alters the amount of circulating E2 as the pathway that produces E2 is inhibited, resulting in compromised reproductive functions, such as ovulation. Considering both current and previous results, it can be assumed that the repression of heat stress on steroid hormone secretion could be due to the reduction in cell proliferation and the disruption of steroid hormone synthesis. However, the mechanism behind the transient elevation of E2 levels after 12 h is still unknown; we inferred that GCs may have a compensatory effect and temporarily increased the secretion of E2 after heat stress (between 12 to 24 h), but continuous high temperature destroyed the steroid hormone synthesis function of GCs, while the E2 production from the heat-stressed cells was lower than that from the normal cells.

It is well documented that, in response to various stressors, HSP family genes will be activated in cells. In our present study, the expression of some HSP family genes, such as HSP70 and HSP90, was significantly increased in heat-stress-challenged GCs. HSP70 is the most pronounced and is involved in GC functions; high temperatures can stimulate the transcription of HSP70 in ovarian follicular GCs [19,31,36]. HSP70 levels in the peripheral blood of laying hens also showed a marked increase when exposed to a high-temperature environment [37]. In addition, in porcine GCs, the HSP70 reduced the activity of FSHR and CYP19A1 promoters, along with the decreased synthesis of estradiol [38]. HSP70 is related to the inhibition of hormone-sensitive steroidogenesis and can hamper cholesterol translocation to the mitochondrial cytochrome P450scc [39]. Under heat stress, HSP synthesis may be an adaptive cellular response that helps maintain cellular homeostasis.

Actually, in the context of laying hens, although studies have shown the altered impact of heat stress on steroid hormone synthesis in vivo [40], the influence of heat stress on steroid hormone synthesis in vitro has never been researched. In addition, tests on the action of heat stress on steroidogenic gene expression in vitro were previously only conducted in ducks [24]. Therefore, our study is the first to show the effects of heat stress both on steroid hormone synthesis and the expression of the related genes in hen follicular GCs in vitro. Our results verified the results in vivo showing that heat stress elevated P4 concentration but decreased E2 concentration in the large yellow follicular fluid [14] and clarified its molecular mechanism by steroidogenic gene expression.

## 5. Conclusions

The above results can be summed up by saying that laying hens exposed to high environmental temperatures exhibit impaired laying performance. Follicular GCs cultured in vitro are sensitive to the effects of high culture temperature, showing an increase in apoptosis and cellular ultrastructural changes. Heat stress elevated P4 secretion by increasing the expression of StAR, CYP11A1, and 3β-HSD. The results also demonstrated that estradiol synthesis in GCs was inhibited under heat stress through repressing FSHR and CYP19A1 expression. The upregulation of HSP70 under heat stress was also observed. Collectively, laying hens exposed to high temperatures showed damage to follicular GCs and egg production. This study provides a molecular mechanism for the abnormal laying performance of laying hens exposed to heat stress. To the best of our knowledge, this is the first study to demonstrate in vitro the effects of heat stress on steroidogenic gene expression and steroid hormone synthesis in hen follicular GCs.

## Figures and Tables

**Table 1:** The primer sequences for qPCR.

Genes	Accession Number	Primer Sequence (5′–3′)	Fragment Size (bp)
β-actin	NM_205518.2	F: TGTCCCTGTATGCCTCTGGTR: GGGCACCTGAACCTCTCATT	355
HSP70	NM_001006685.1	F: CGGGCAAGTTTGACCTAAR: TTGGCTCCCACCCTATCTCT	250
HSP90	NM_001109785.2	F: AGTCCCAGTTCATTGGCTACR: TCCAGTCATTGGTGAGGCT	324
CYP11A1	NM_001001756.1	F: ACCGTGACTACCGCAACAAGR: AGGCCTCCCCTGTCTTGA	54
3β-HSD	NM_205118.1	F: GCCAAAGAGGAGCAAACCAGAGR: TCCAGCAGTAAGCGAACGATCC	104
StAR	NM_204686.3	F: CGCTGCCATCTCCTACCAACACAGR: GACATCTCCATCTCGCTGAAGG	197
FSHR	NM_205079.2	F: ATGTCTCCGGCAAAGCAAGAR: AACGACTTCGTTGCACAAGC	147
CYP17A1	NM_001001901.3	F: CCACTACCCTGAGGTCCAGAR: GTATTCCCCGATGCTGGTGT	196
CYP19A1	NM_001001761.4	F: CTCGGGGCTGTGTAGGAAAGR: TGTCTGTACTCTGCACCGTC	86

## Data Availability

Data are contained within the article.

## Supplementary Materials

The following supporting information can be downloaded at: https://www.mdpi.com/article/10.3390/ani12111467/s1. Figure S1: Characterization of cultured follicular GCs from laying hens and FSHR fluorescence immunostaining. Table S1: Ingredients and nutrient composition of the experimental basal diet (as fed basis) fed to laying hens.

## References

[1] Mignon-Grasteau S., Moreri U., Narcy A., Rousseau X., Rodenburg T.B., Tixier-Boichard M., Zerjal T. (2015). Robustness to chronic heat stress in laying hens: A meta-analysis. Poult. Sci..

[2] Lara L.J., Rostagno M.H. (2013). Impact of heat stress on poultry production. Animals.

[3] Hu J., Xiong Y., Gates R.S., Cheng H.W. (2021). Perches as Cooling Devices for Reducing Heat Stress in Caged Laying Hens: A Review. Animals.

[4] He S.P., Arowolo M.A., Medrano R.F., Li S., Yu Q.F., Chen J.Y. (2018). Impact of Heat Stress on Poultry Production. World. Poult. Sci. J..

[5] Rozenboim I., Tako E., Gal-Garber O., Proudman J.A., Uni Z. (2007). The effect of heat stress on ovarian function of laying hens. Poult. Sci..

[6] Ebeid T.A., Suzuki T., Sugiyama T. (2012). High temperature influences eggshell quality and calbindin-D28k localization of eggshell gland and all intestinal segments of laying hens. Poult. Sci..

[7] Emery D.A., Vohra P., Ernst R.A., Morrison S.R. (1984). The effect of cyclic and constant ambient temperatures on feed consumption, egg production, egg weight, and shell thickness of hens. Poult. Sci..

[8] Mashaly M.M., Hendricks G.L., Kalama M.A., Gehad A.E., Abbas A.O., Patterson P.H. (2004). Effect of heat stress on production parameters and immune responses of commercial laying hens. Poult. Sci..

[9] Barrett N.W., Rowland K., Schmidt C.J., Lamont S.J., Rothschild M.F., Ashwell C.M., Persia M.E. (2019). Effects of acute and chronic heat stress on the performance, egg quality, body temperature, and blood gas parameters of laying hens. Poult. Sci..

[10] Kim D.H., Lee Y.K., Lee S.D., Kim S.H., Lee S.R., Lee H.G., Lee K.W. (2020). Changes in production parameters, egg qualities, fecal volatile fatty acids, nutrient digestibility, and plasma parameters in laying hens exposed to ambient temperature. Front. Vet. Sci..

[11] Kim D.H., Lee Y.K., Kim S.H., Lee K.W. (2021). The Impact of Temperature and Humidity on the Performance and Physiology of Laying Hens. Animals.

[12] Hu S.Q., Zadworny D. (2017). Effects of nonglycosylated and glycosylated prolactin on basal and gonadotropin-stimulated steroidogenesis in chicken ovarian follicles. Domest. Anim. Endocrinol..

[13] Li G.M., Liu L.P., Yin B., Liu Y.Y., Dong W.W., Gong S., Zhang J., Tan J.H. (2020). Heat stress decreases egg production of laying hens by inducing apoptosis of follicular cells via activating the FasL/Fas and TNF-α systems. Poult. Sci..

[14] Johnson A.L., Solovieva E.V., Bridgham J.T. (2002). Relationship between steroidogenic acute regulatory protein expression and progesterone production in hen granulosa cells during follicle development. Biol. Reprod..

[15] Li Z., Johnson A.L. (1993). Regulation of P450 cholesterol side-chain cleavage messenger ribonucleic acid expression and progesterone production in hen granulosa cells. Biol. Reprod..

[16] Young J.M., McNeilly A.S. (2010). Theca: The forgotten cell of the ovarian follicle. Reproduction.

[17] Jiang S., Mohammed A.A., Jacobs J.A., Cramer T.A., Cheng H.W. (2020). Effect of synbiotics on thyroid hormones, intestinal histomorphology, and heat shock protein 70 expression in broiler chickens reared under cyclic heat stress. Poult. Sci..

[18] Zhao Q., Xue W., Zhang S., Guo Y., Li Y., Wu X., Huo S., Li Y., Li C. (2022). The functions of Patchouli and Elsholtzia in the repair of hen follicular granular cells after heat stress. Poult. Sci..

[19] Sun N., Zhang Y., Hou Y., Yi Y., Guo J., Zheng X., Sun P., Sun Y., Khan A., Li H. (2020). Effects of Osthole on Progesterone Secretion in Chicken Preovulatory Follicles Granulosa Cells. Animals.

[20] Sun L., Lamont S.J., Cooksey A.M., McCarthy F., Tudor C.O., Vijay-Shanker K., DeRita R.M., Rothschild M., Ashwell C., Persia M.E. (2015). Transcriptome Response to Heat Stress in a Chicken Hepatocellular Carcinoma Cell Line. Cell Stress Chaperon..

[21] Mackei M., Molnár A., Nagy S., Pál L., Kővágó C., Gálfi P., Dublecz K., Husvéth F., Neogrády Z., Mátis G. (2020). Effects of Acute Heat Stress on a Newly Established Chicken Hepatocyte—Nonparenchymal Cell Co-Culture Model. Animals.

[22] Yang C., Huang X.B., Chen S.J., Li X.J., Fu X.L., Xu D.N., Tian Y.B., Liu W.J., Huang Y.M. (2021). The effect of heat stress on proliferation, synthesis of steroids, and gene expression of duck granulosa cells. Anim. Sci. J..

[23] Qu X., Guo S., Yan L., Zhu H., Li H., Shi Z. (2020). TNFα-Erk1/2 signaling pathway-regulated SerpinE1 and SerpinB2 are involved in lipopolysaccharide-induced porcine granulosa cell proliferation. Cell. Signal..

[24] Zhu H., Shao X., Chen Z., Wei C., Lei M., Ying S., Yu J., Shi Z. (2017). Induction of out-of-season egg laying by artificial photoperiod in Yangzhou geese and the associated endocrine and molecular regulation mechanisms. Anim. Reprod. Sci..

[25] Zhu H., Chen Z., Shao X., Yu J., Wei C., Dai Z., Shi Z. (2017). Reproductive axis gene regulation during photostimulation and photorefractoriness in Yangzhou goose ganders. Front. Zool..

[26] Guo B., Qu X., Chen Z., Yu J., Yan L., Zhu H. (2022). Transcriptome analysis reveals transforming growth factor-β1 prevents extracellular matrix degradation and cell adhesion during the follicular-luteal transition in cows. J. Reprod. Develop..

[27] Livak K.J., Schmittgen T.D. (2001). Analysis of relative gene expression data using real-time quantitative PCR and the 2−ΔΔCT method. Methods.

[28] Oguntunji O.M., Alabi A.O. (2010). Influence of high environmental temperature on egg production and shell quality, a review. World. Poult. Sci. J..

[29] Rubio M., Alves L., Viana G., Benevides V.P., Junior A.B. (2020). Heat stress impairs egg production in commercial laying hens infected by fowl typhoid. Avian Pathol..

[30] Vandana G.D., Sejian V., Lees A.M., Pragna P., Maloney S.K. (2021). Heat stress and poultry production: Impact and amelioration. Int. J. Biometeorol..

[31] Li L., Wu J., Luo M., Sun Y., Wang G. (2016). The effect of heat stress on gene expression, synthesis of steroids, and apoptosis in bovine granulosa cells. Cell Stress Chaperon..

[32] Stocco D.M. (2001). StAR protein and the regulation of steroid hormone biosynthesis. Annu. Rev. Physiol..

[33] Burris-Hiday S.D., Scott E.E. (2021). Steroidogenic cytochrome P450 17A1 structure and function. Mol. Cell. Endocrinol..

[34] Nteeba J., Sanz-Fernandez M.V., Rhoads R.P., Baumgard L.H., Ross J.W., Keating A. (2015). Heat stress alters ovarian insulin mediated phosphatidylinositol-3 kinase and steroidogenic signaling in gilt ovaries. Biol. Reprod..

[35] Heidarzadehpilehrood R., Pirhoushiaran M., Abdollahzadeh R., Binti Osman M., Sakinah M., Nordin N., Abdul Hamid H. (2022). A Review on CYP11A1, CYP17A1, and CYP19A1 Polymorphism Studies: Candidate Susceptibility Genes for Polycystic Ovary Syndrome (PCOS) and Infertility. Genes.

[36] Sirotkin A.V., Bauer M. (2011). Heat shock proteins in porcine ovary: Synthesis, accumulation and regulation by stress and hormones. Cell Stress Chaperon..

[37] Maak S., Melesse A., Schmidt R., Schneider F., Von Lengerken G. (2003). Effect of long-term heat exposure on peripheral concentrations of heat shock protein 70 (Hsp70) and hormones in laying hens with different genotypes. Br. Poult. Sci..

[38] Li H., Guo S., Cai L., Ma W., Shi Z. (2017). Lipopolysaccharide and heat stress impair the estradiol biosynthesis in granulosa cells via increase of HSP70 and inhibition of smad3 phosphorylation and nuclear translocation. Cell. Signal..

[39] Khanna A., Aten R.F., Behrman H.R. (1994). Heat shock protein induction blocks hormone-sensitive steroidogenesis in rat luteal cells. Steroids.

[40] Attia Y.A., El-Hamid A.E., Abedalla A.A., Berika M.A., Al-Harthi M.A., Kucuk O., Sahin K., Abou-Shehema B.M. (2016). Laying performance, digestibility and plasma hormones in laying hens exposed to chronic heat stress as affected by betaine, vitamin C, and/or vitamin E supplementation. Springerplus.

