# Supplementary material for: Effect of Heat Stress on Egg Production, Steroid Hormone Synthesis, and Related Gene Expression in Chicken Preovulatory Follicular Granulosa Cells
Source: Animals (Basel). 2022 Jun 5;12(11):1467. doi: 10.3390/ani12111467 (PMC9179568; doi:10.3390/ani12111467)
Supplement: Supplementary file 1 [file animals-12-01467-s001.zip › animals-1719428-SI.pdf]

Table S1. Ingredients and nutrient composition of the experimental basal diet (as fed basis) fed to laying hens

Ingredients	Content (%)	Calculated or analyzed composition	
Corn	57	ME (MJ/kg) [2]	11.2
Soybean meal, 43% crude protein	22	crude protein	17.50
Corn gluten meal	5	Ether extract	6.32
Rice bran	3	Lysine	0.78
Limestone	8	Methionine	0.54
Premix [1]	3	Calcium	3.52
NaCl	0.4	Total phosphorus	0.48
Calcium hydrophosphate	1.6	Available phosphorus	0.29
Total	100.00		

[1] The premix provides the following per kg diet: vitamin A, 7000 IU; vitamin D3, 2500 IU; vitamin E, 36 mg; vitamin K, 32 mg; vitamin B12, 0.025 mg; vitamin B2, 5.6 mg; vitamin B6, 4 mg; vitamin B12, 0.025 mg; nicotinic acid, 38 mg; folic acid, 1.1 mg; calcium pantothenate, 10 mg; biotin, 0.16 mg; Cu, 10 mg; Fe, 80 mg; Mn, 100 mg; Zn, 60 mg; I, 0.55 mg; and Se, 0.12 mg.[2] ME, metabolic energy, calculated values.

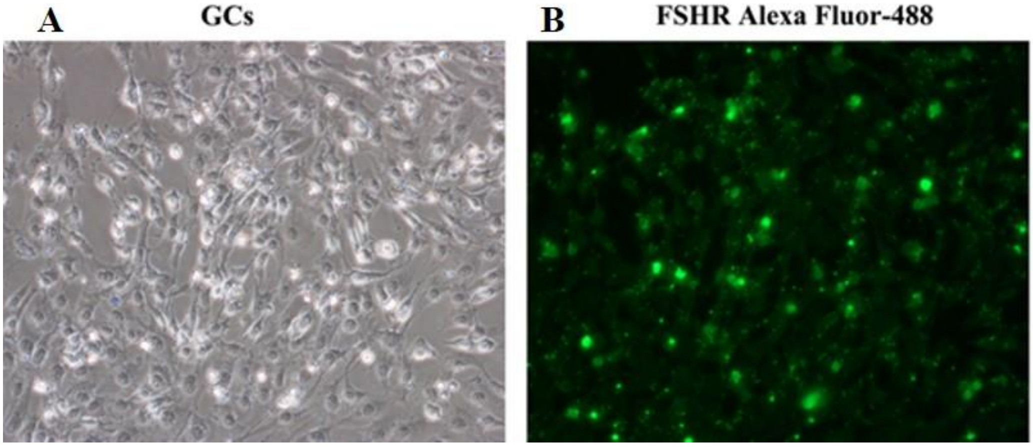

Supplementary Figure S1. Characterization of cultured follicular granulosa cells from laying hens and FSHR fluorescence immunostaining. A: The granulosa cells cultured for 48 h of post isolation. B:Immunoblotting for anti-FSHR cytoplasm (green).
